# Editorial: Economic evaluation in evidence-based criminal justice contexts

**DOI:** 10.3389/fpsyg.2024.1429063

**Published:** 2024-07-18

**Authors:** Susan Giles, Siddhartha Bandyopadhyay, Karen Shalev, Matthew Manning

**Affiliations:** ^1^Department of Psychology, University of Liverpool, Liverpool, United Kingdom; ^2^Department of Economics, University of Birmingham, Birmingham, United Kingdom; ^3^School of Criminology and Criminal Justice, University of Portsmouth, Portsmouth, United Kingdom; ^4^Department of Social and Behavioural Science, City University of Hong Kong, Kowloon, Hong Kong SAR, China

**Keywords:** evidence-based policing, economic evaluation, cost benefit, cost utility, cost effectiveness, costs of crime, social value

In times of economic austerity, criminal justice agencies are required to make evidence-based decisions that yield optimal return. The aim of this inter-disciplinary special issue is to showcase economic analysis taking place in policing and criminal justice contexts, using both established and innovative techniques. It is hoped that this will contribute a robust evidence base alongside demonstrating innovation in economic methodologies that could be beneficial to other researchers. Four quality publications were received, demonstrating innovative economic practices in estimating treatment effects or directing resources to high-risk individuals.

## Understanding the effectiveness and cost effectiveness of a perpetrator intervention programme

Domestic violence is a pervasive phenomenon for which a number of within and across generational negative impacts are assessed. Yet the evidence base around what works is still patchy and economic analysis of interventions is particularly limited. Karavias et al. consider the impact of a so-called “batterer intervention programme” (BIP) called CARA (Cautioning and Relationship Abuse) and find a strong reduction in reoffending among those who attended the programme across two police force areas with very different socio-economic and demographic characteristics. Their impact evaluation naturally leads to an economic evaluation quantifying the benefit achieved in monetary terms. It indicates the monetised benefit of the intervention ranges from £2.75–11.1 per pound spent. This strongly suggests that CARA will deliver benefits by reducing reoffending and be economically efficient if rolled out across more police forces. The use of machine learning methods to identify the most important variables that determine treatment selection and being able to use boundedness tests to show that unobservable factors would need to have a dramatic impact to invalidate the results provide robustness to the analysis.

## Incorporating impact heterogeneity into cost-benefit analysis

Traditional cost-benefit analyses (CBA) rely on average treatment effects and do not consider contextual factors that moderate outcomes for community sub-groups. Existing gains, as a result, may disproportionately target and benefit certain subgroups. This is problematic for criminal justice interventions where poverty and access to justice may influence outcomes. Manning and colleagues consider how justice processes treat different groups and whether CBA can be enhanced by the inclusion of such heterogeneity Manning et al. (a, b). Drawing upon similar past research, an economic framework is suggested including quantile treatment effects and a range of moderators (e.g., ethnicity, gender, latent constructs, exclusion, and governance). The enhanced CBA APP is demonstrated using existing data from a school-based intervention in Australia. Future developments, including machine learning, are then considered. The current research offers considerable methodological innovation. By moving away from average treatment effects and overall societal benefit, the enhanced CBA APP potentially improves the accuracy of resource allocation so that finite resources are directed more equitably. It can help achieve maximum economic and social outcomes whilst targeting unequal treatment and outcomes for vulnerable and excluded social groups.

## Offense prioritization in high-volume, high-harm crimes

Giles et al. discuss the pervasive risk or harm posed by online child sexual abuse, which strains law enforcement's ability to respond effectively. Whilst prioritization methods exist for individuals with experience of offline offenses, there is a lack of focus on online-only offenses (OOCSA), partly due to ambiguity regarding victim harm and online offending's contribution to it. Giles et al. produce a narrative review to address this gap, identifying five themes from existing literature: problems defining OOCSA, normalizing online harm, OOCSA grooming processes, comparisons with offline abuse, and the mechanisms between OOCSA and harm. They suggest factors like shame, reach of abuse, image permanence, victim vulnerability, and social support could guide prioritization strategies. Drawing upon original police data, crime reports and surveys they estimate the economic burden of OOCSA in England and Wales. Adapting UK Home Office figures to OOCSA they establish lifetime costs (£7.4 million based on police reports), scaling up to consider undetected crimes (£59.6 million) and national prevalence (£1.4 billion from self-report surveys). This research highlights the potential for economic models in understanding and addressing novel areas like OOCSA, providing insights for future researchers and law enforcement to develop evidence-led tools and strategies.

## An economic evaluation of restorative justice post sentence in England and Wales

Participation in restorative justice interventions post-sentence has been shown to reduce reoffending and mitigate harm to victims. Investment in, and access to, restorative justice remains limited in England and Wales. Focusing on direct and indirect restorative justice interventions for victims and offenders post-sentence in England and Wales, Jones et al. developed a model to estimate the social benefit–cost ratio of restorative justice, as well as the direct financial return to the criminal justice system. Their estimates suggest that increasing the proportion of eligible cases referred for a restorative justice intervention from 15 to 40% could be associated with an increase in investment of £5 m, and benefits to the criminal justice system totaling £22 m, implying a net saving of £17 m. The economic case for investment in restorative justice centers on identifying offenders with a high risk of offending and enabling them to participate in an intervention that has been repeatedly demonstrated to help them to change their behavior. The study can help advance policymakers' understanding of the value of restorative justice as well as how to harness this value to benefit victims, offenders and society.

## Summary contributions

Each paper has contributed new knowledge that will enhance the rigor and external validity of economic models. The fact that we received only four papers for this special issue, despite having an extended timeline, is a testament to the time it takes to produce high-quality economic evaluations. Its relative scarcity is sometimes further compromised by data issues and a reluctance to venture into an area that is not set up to appropriately measure the economic costs and benefits. The papers however demonstrate that with advances in methodology, if appropriate data were routinely collected, robust economic analysis can be undertaken providing robust evidence on the effective use of scarce resources. We hope that readers can derive benefits from the innovations presented here.

## Author contributions

SG: Writing – original draft, Writing – review & editing. SB: Writing – original draft, Writing – review & editing. KS: Writing – original draft, Writing – review & editing. MM: Writing – original draft, Writing – review & editing.

